# *Staphylococcus aureus*-Induced Necroptosis Promotes Mitochondrial Damage in Goat Endometrial Epithelial Cells

**DOI:** 10.3390/ani12172218

**Published:** 2022-08-29

**Authors:** Yanyan Yi, Kangkang Gao, Pengfei Lin, Huatao Chen, Dong Zhou, Keqiong Tang, Aihua Wang, Yaping Jin

**Affiliations:** Key Laboratory of Animal Biotechnology of the Ministry of Agriculture, College of Veterinary Medicine, Northwest A&F University, Yangling 712100, China

**Keywords:** *Staphylococcus aureus*, goat endometrial epithelial cells, necroptosis, ion dysregulation, mitochondrial damage

## Abstract

**Simple Summary:**

The death of endometrial cells induced by bacterial infections can result in damage to the endometrial function. In this study, we investigated the potential role of necroptosis in *Staphylococcus aureus*-induced goat endometrial epithelial cell (gEEC) death. We found that *S. aureus* induced gEECs RIPK1/RIPK3/MLKL-mediated necroptosis, triggered mainly by membrane disruption and ion imbalance. Moreover, gEEC necroptosis contributed to the regulation of reactive oxygen species generation and mitochondrial damage. These provide evidence of the involvement of necroptosis in the *S. aureus*-induced gEEC death.

**Abstract:**

Endometrial cell death is induced by bacterial infection, resulting in damage to the physical barriers and immune function. An in-depth understanding of the mechanisms of endometrial epithelial cell necroptosis might provide new insights into the treatment of uterine diseases. In the present study, we investigated the effect of *Staphylococcus aureus* on goat endometrial epithelial cell (gEEC) necroptosis, and the underlying molecular mechanism. We found that *S. aureus* induced significant necroptosis in gEECs by increasing the expression of key proteins of the RIPK1/RIPK3/MLKL axis; importantly, this effect was alleviated by inhibitors of RIPK1, RIPK3, and MLKL. Moreover, we found that the main triggers of gEEC necroptosis induced by *S. aureus* were not the toll-like receptors (TLRs) and tumor necrosis factor receptor (TNFR), but membrane disruption and ion imbalance. Moreover, we observed a significant decrease in the mitochondrial membrane potential, indicating mitochondrial damage, in addition to increased cytochrome c levels and reactive oxygen species (ROS) generation in *S. aureus*-infected gEECs; these, effects were also suppressed by the inhibitors of RIPK1, RIPK3, and MLKL. Taken together, these data revealed the molecular mechanism of *S. aureus*-induced gEEC necroptosis and provided potential new targeted therapies for clinical intervention in bacterial infections.

## 1. Introduction

Endometritis is a common disease in ruminants and is caused mainly by a bacterial infection in the uterus after parturition [1,2]. Endometritis can cause prolonged uterine involution and poor reproductive outcomes, which in turn leads to increased culling rates and serious economic losses to the livestock farming industry [3,4,5]. New methods for the effective prevention and control of postpartum endometritis are urgently needed; however, their development depends on a clear understanding of the mechanism of interaction between endometrial cells and pathogenic bacteria. Inflammatory damage to endometrial epithelial cells (EECs) is critical in the endometrial inflammatory cascade response and pathological process [6]. Since multiple forms of cell death and regulatory pathways are involved in the inflammatory response, elucidation of the cell death mechanisms involved in this process is vitally important.

Pathological histological examination of acute endometritis tissue samples revealed degeneration and necrosis of the endometrial cells, suggesting that necrosis may be involved in the inflammatory response process associated with this condition [7]. However, whether the necrosis in acute endometritis could regulate by proteins remain to be clarified. Of particular note, multiple forms of apoptosis and necrosis have been recognized, including a form of programmed necrosis known as necroptosis [8]. Importantly, the discovery of necroptosis suggested that “passive, unregulated” necrosis can be at least partially disrupted and blocked, such as apoptosis, and has aroused widespread interest and enthusiasm among researchers. Multiple necroptosis-related proteins have been identified to be involved to date. In general, the initiation stage of necroptosis is thought to be triggered by TNF receptor 1/2 (TNFR1/2). The receptor-interacting serine/threonine-protein (RIP) kinase family members RIPK1 and RIPK3, which are essential for signal transduction pathways in necroptosis, interact to form a RIPK1-RIPK3 complex, also known as the necrosome, mediated by the RIP homotypic interaction motif (RHIM) domain. The necrosome recruits and phosphorylates the substrate mixed lineage kinase domain-like protein (MLKL). This results in the translocation of phosphorylated MLKL (P-MLKL) to the cell membrane where large MLKL oligomers are formed, which cause cell rupture and release of the cytoplasmic components [9]. Necroptosis can also be initiated by other molecules, such as toll-like receptors (TLRs), nucleotide-binding and oligomerization domain-like receptors (NLRs), Z-DNA binding protein 1 (ZBP1; also known as DAI), and interferon (IFN) [10]. Studies have also shown that a variety of pathogens induce host cell necroptosis. The excessive release of TNF induced by *Mycobacterium tuberculosis* causes tissue damage and organ failure via activating the RIPK1/RIPK3/MLKL-dependent necrotic pathway [11]. In addition, infection of macrophages with *Legionella pneumophila* leads to rapid histone B-dependent necroptosis and the release of damage-associated molecular patterns (DAMPs) [12]. *Brucella abortus* has been reported to induce caspase 2-mediated apoptosis and necrosis in mouse macrophages [13]. During *Salmonella typhimurium* infection, macrophages deficient in the receptor for type I interferons or RIPK3 showed a greater survival in mice [14]. Similarly, the induction of necroptosis through RIPK1/RIPK3/MLKL signaling is a major consequence of *S. aureus* infection in alveolar macrophages and keratinocyte cell lines and can be prevented by RIPK3 depletion or inhibition of RIPK1 and MLKL [15,16]. Moreover, the pore-forming toxins (PFTs) secreted by *S. aureus*, *Streptococcus pneumoniae*, and *Staphylococcus marcescens* have been shown to induce necroptotic cell death, which can be arrested by necroptosis inhibitors [17,18]. Hence, necroptosis may be an important pathway by which host cells respond to pathogen infection, and targeted regulation strategies may yield effective measures for the management of infectious diseases with a necrotic phenotype. Importantly, unlike macrophages, lung epithelial cells, and keratinocytes, the impact of necroptosis on endometrial epithelium cells during bacterial endometritis has not been specifically examined.

Studies of the postpartum uterus have shown that bacteria, such as *Escherichia coli*, are common causes of the release of inflammatory factors in EECs. *Trueperella pyogenes* causes severe cell death, though stromal cells are more sensitive to this infection than EECs. *S. aureus* is a major cause of severe inflammatory EECs damage and shedding [19]. Previous studies have demonstrated that *S. aureus* can activate necroptosis, leading to the loss of specific types of cells, and promoting pathological changes in the host cells by disrupting critical immunomodulatory functions [20,21]. Based on the morphology of EECs damage, we speculated that necroptosis plays a role in the cell damage in endometritis caused by *S. aureus*.

Therefore, in this study, we aimed to unravel the effect of necroptosis on bacterial-induced death of EECs and the underlying mechanism. We demonstrated that significant necroptosis occurs in acute endometritis caused by *S. aureus*, in which ion imbalance is the main cause. We also confirmed that necroptosis is involved in promoting mitochondrial damage and reactive oxygen species (ROS) generation. This finding provides new information that can be used to refine our understanding of the mechanism of EECs damage associated with pathogenic bacterial infections and provides ideas for the development of novel targeted drugs for effective prevention and control of postpartum endometritis.

## 2. Materials and Methods

### 2.1. Cells and Bacterial Strains

The immortalized goat endometrial epithelial cell line conserved in our laboratory was cultured in Dulbecco’s Modified Eagle Medium (DMEM)/F12 (HyClone, Logan, UT, USA) supplemented with 10% fetal bovine serum (FBS) (ZETA Life, Arcadia, CA, USA), and 1% penicillin–streptomycin (Solarbio Life Science, Beijing, China) at 37 °C in a humidified incubator under 5% CO_2_. *S. aureus* (ACCC 01011) was a gift from the Veterinary Public Health and Livestock Product Safety Laboratory of Northwest A&F University and was recovered in Luria–Bertani (LB) solid medium at 37 °C for 12 h. When indicated, *S. aureus* was resuspended in phosphate-buffered saline (PBS) at the required density.

### 2.2. Bacterial Infection

For infection experiments, *S. aureus* was added to cells at a multiplicity of infection (MOI) of 10 or 100 for up to 6 h. For inhibition experiments, the cells were pretreated 1 h before infection with Necrostatin 2 recamate (Nec-1s, T7504) GSK’872 (T4074), Necrosulfonamide (NSA, T7129), TLR-IN-C34 (T8503), R7050 (T4637), Acetylcysteine (NAC, T0875), and rotenone (T2970) (TargetMol Biotech, Boston, MA, USA), glycine (1275, Biofroxx, Einhausen, Germany), and ionomycin (S1672, Beyotime Biotech, Beijing, China) at indicated concentrations.

### 2.3. Lactate Dehydrogenase (LDH) Release

LDH release was determined using a CytoTox96^®^ non-radioactive cytotoxicity assay kit (Promega Biotech, Madison, WI, USA) according to the manufacturer’s instructions. Briefly, after centrifugation at 1500 rpm/min for 5 min, the supernatant was collected and 50 μL was added to a 96-well plate, followed by the addition of CytoTox 96^®^ Reagent. The cells were then incubated in the absence of light for 30 min at room temperature. The reaction was terminated by adding 50 μL of stop solution, and the OD value was recorded at 490 nm using a microplate reader.

### 2.4. Cell Death Detection

Live and dead cells were distinguished using a Calcein/PI staining assay kit (Beyotime Biotech, Beijing, China) following the manufacturer’s instructions. Briefly, after washing the cells twice with PBS, Calcein/PI assay working solution was added and the cells were incubated in the absence of light for 30 min at 37 °C before observation under a fluorescence microscope (Axio Observer, Carl Zeiss AG, Oberkochen, Germany).

### 2.5. Transmission Electron Microscopy (TEM)

After infection with *S. aureus* at 10 MOI for 4 h, the morphological changes of cells were observed by TEM performed by ServiceBio (Wuhan, China).

### 2.6. Western Blot Analysis

After washing twice with PBS, total proteins were extracted from cells by the addition of 100 μL of lysis buffer to each well. After lysis on ice for 30 min, the cells were centrifuged at 12,000× rpm/min for 15 min at 4 °C. The supernatant was then collected, and the protein concentration was assayed using a BCA protein concentration assay kit (Beyotime Biotech, Beijing, China). Proteins were separated by SDS-PAGE and then transferred to a PVDF membrane. After being blocked with 5% non-fat dry milk for 2 h at room temperature, the membranes were subsequently incubated with the following primary antibodies at 4 °C overnight: RIPK1 (ab125072), P-RIPK3 (ser227, ab209384), P-MLKL (ser358, ab187091) (Abcam, Cambridge, UK), RIPK3 (sc-374639) and MLKL (sc-293201) (Santa Cruz Biotechnology, CAUSA), β-actin (Proteintech, Wuhan, China). After three washes with TBST, the HRP-conjugated secondary antibody (Zhonghuihecai, Shaanxi, China) was added and incubated at room temperature for 1 h. Finally, ECL luminescent solution was used to detect the bands of target proteins, and grayscale quantification analysis was performed using Image J software.

### 2.7. Indirect Immunofluorescence Assay (IFA)

After infection with *S. aureus* for 4 h, gEECs were fixed with 4% paraformaldehyde for 20 min and then permeabilized with 0.3% Triton X-100 for 5 min at room temperature. After blocking with 3% bovine serum albumin (BSA) for 2 h, the cells were incubated with mouse anti-P-MLKL (Ser358) antibody (bsm-33331M, Bioss, Wuhan, China) overnight at 4 °C. After three washes with PBS, the cells were then incubated with CoraLite594-conjugated goat anti-mouse IgG (H+L) (SA00013-3, Proteintech, Wuhan, China) for 1 h, followed by staining with DAPI for 15 min. Finally, captured laser scanning confocal microscopy (LSM510 META, Zeiss, Germany) was performed to capture images.

### 2.8. JC-1 Staining

Mitochondrial membrane potential (mitochondrial membrane potential) was determined using a JC-1 staining assay kit according to the manufacturer’s protocols (GOYOO Biotech, Nanjing, China). Briefly, after treatment, the cells were incubated with a JC-1 stain working solution at 37 °C for 20 min in the absence of light. The cells were then washed twice with JC-1 staining buffer (1×) and the nuclei were stained with DAPI. Finally, cells were observed under a fluorescence microscope, and images were captured.

### 2.9. Cytochrome C Assay

An enzyme-linked immunosorbent assay (ELISA) kit (Mbbiology, Nanjing, China) was used to detect the cytochrome C (cytochrome C) levels in cells. Briefly, the cells were lysed using RIPA lysis buffer (Beyotime Biotech, Beijing, China), and the supernatant was collected. The protein concentration was determined using the BCA protein assay kit (Beyotime Biotech, Beijing, China). For the ELISA assay, 50 μL of standard and sample were added to each well, mixed with 100 μL of enzyme labeling solution, and then incubated for 1 h at 37 °C. After five washes with washing solution, 50 μL of solution A and B were added, respectively, and incubated for 15 min at 37 °C. Finally, 50 μL of termination solution was added, and the OD values at 450 nm were measured using a microplate reader. The cytochrome C content was calculated based on the standard curve and protein concentration.

### 2.10. ROS Assay

ROS levels were measured using the fluorescent probe DCFH-DA (Beyotime Biotech, Beijing, China). After infection of cells with *S. aureus*, the supernatant was removed and the cells were incubated with DCFH-DA diluted in a serum-free medium. After incubation for 20 min at 37 °C, the cells were washed three times with serum-free medium and observed and photographed under a fluorescence microscope at 488 nm.

### 2.11. Calcium Ion Imaging

Fluo-4 AM (Beyotime Biotech, Beijing, China) was used to detect intracellular Ca^2+^ concentration. Cells were washed three times before fluorescent probe loading 2 μM of Fluo-4 AM working solution for 20 min at 37 °C for fluorescent probe loading. Subsequently, the cells were washed three times with PBS and observed under a fluorescence microscope to determine changes in the intracellular Ca^2+^ levels.

### 2.12. Statistical Analysis

Data were analyzed using GraphPad Prism^TM^ 7 software (GraphPad Software Inc., La Jolla, CA, USA), one-way analysis of variance (ANOVA) was used for multiple-group comparisons, and a t-test was applied for the two-group comparisons. Data were presented as mean ± standard deviation (SD) and *p* < 0.05 was considered to indicate statistical significance. All experiments were repeated three times.

## 3. Results

### 3.1. S. aureus Induces gEEC Necrotic Cell Death

**Figure 1:**
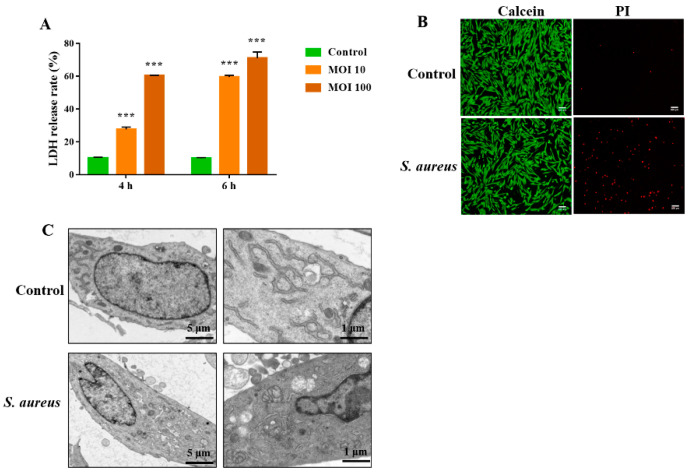
Effect of *S. aureus* on gEEC necrotic cell death. (**A**) gEECs were infected with *S. aureus* (10 and 100 MOI) for 2 and 4 h respectively, then the cytotoxicity was detected using a CytoTox96^®^ non-radioactive cytotoxicity assay kit. (**B**) gEECs were infected with *S. aureus* (MOI 10) for 4 h, calcein/PI staining was performed to assay the necrotic rate in *S. aureus*-infected gEECs. (**C**) TEM analysis of *S. aureus*-infected gEECs. Typical necrotic structures are clearly visualized: organelle swelling, cytoplasmic vacuolization, and chromatin agglutination. *** *p* < 0.001 vs. control group.

Previous studies revealed that necroptosis is involved in the death of *S. aureus*-infected macrophages and lung epithelial cells [15,16]. However, it is unclear whether *S. aureus* induces endometrial epithelium cell necroptosis. Therefore, in this study, we investigated the ability of *S. aureus* to induce necroptosis in the immortalized gEECs lines and its underlying mechanism in the present study. The cells were infected with *S. aureus* at 10 MOI and 100 for 4 h and 6 h, respectively, then the cytotoxicity was detected using a CytoTox96^®^ non-radioactive cytotoxicity assay kit. As shown in Figure 1A, *S. aureus* induced significant gEEC death in a dose-dependent manner as indicated by the increased release of LDH. Next, after infection with *S. aureus* (10 MOI) for 4 h, gEECs were subjected to Calcein/PI staining and fluorescence microscopy analysis. As shown in Figure 1B, the increased number of PI-positive cells showed that *S. aureus* induced significant necrotic death of gEECs. As shown in Figure 1C, necrotic death of gEECs was confirmed at the ultrastructural level, with typical features of necrotic cell death, such as organelle swelling, cytoplasmic vacuolization, and chromatin agglutination observed. These data suggested that necrotic cell death is an important component of *S. aureus*-induced gEEC death.

### 3.2. The RIPK1/RIPK3/MLKL Signaling Pathway Contributes to S. aureus-Induced gEEC Necroptosis

**Figure 2:**
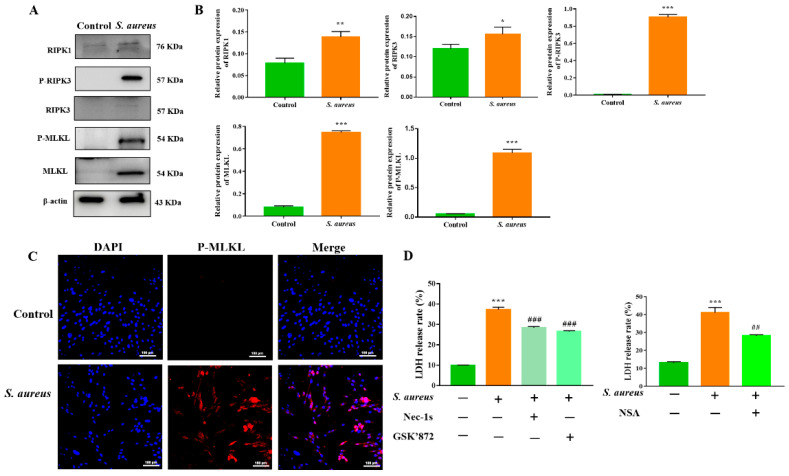
*S. aureus* induces RIPK1/RIPK3/MLKL-mediated necroptosis in gEECs. (**A**) gEECs were infected with *S. aureus* (10 MOI) for 4 h; western blot analysis was performed to detect the expression of the necroptosis-associated protein. (**B**) The band intensity was quantified with the ImageJ program and normalized with β-actin. (**C**) IFA analysis of P-MLKL in *S. aureus*-infected gEECs. (**D**) gEECs were pretreated with the inhibitors of RIPK1, RIPK3, and MLKL (Nec-1s, GSK’872, and NSA) for 2 h, respectively, then infected with 10 MOI *S. aureus* for 4 h. The cytotoxicity was determined using a CytoTox96^®^ non-radioactive cytotoxicity assay kit. * *p* < 0.05, ** *p* < 0.01, *** *p* < 0.001 vs. control group, ## *p* < 0.01, ### *p* < 0.001 vs. *S. aureus* group.

Necroptosis is defined as a regulatory form of programmed cell death. According to multiple models, the RIPK1/RIPK3/MLKL axis is considered to be the classical signaling pathway by which necroptosis is regulated. Hence, we performed a western blot assay to determine the expression of RIPK1, RIPK3, and MLKL in gEECs infected with or without (10 and 100 MOI) *S. aureus* for 4 h. As shown in Figure 2A,B and Figure S1, increased expression of RIPK1, RIPK3, and MLKL proteins, and their phosphorylated forms were detected after *S. aureus* infection. IFA further confirmed that *S. aureus* induced gEEC necroptosis as indicated by the increased expression and localization of P-MLKL in the cell membrane (Figure 2C). Moreover, *S. aureus*-induced gEEC necroptosis was alleviated by pretreatment with necroptotic inhibitors (Nec-1s, GSK’872, NSA), as indicated by the reduction in LDH release (Figure 2D). Taken together, these results clearly indicated that RIPK1/RIPK3/MLKL-mediated necroptosis is an important mode of *S. aureus*-induced gEEC death.

### 3.3. S. aureus-Induced gEEC Necroptosis Is Not Dependent on Death Receptor Signaling and TLR4

**Figure 3:**
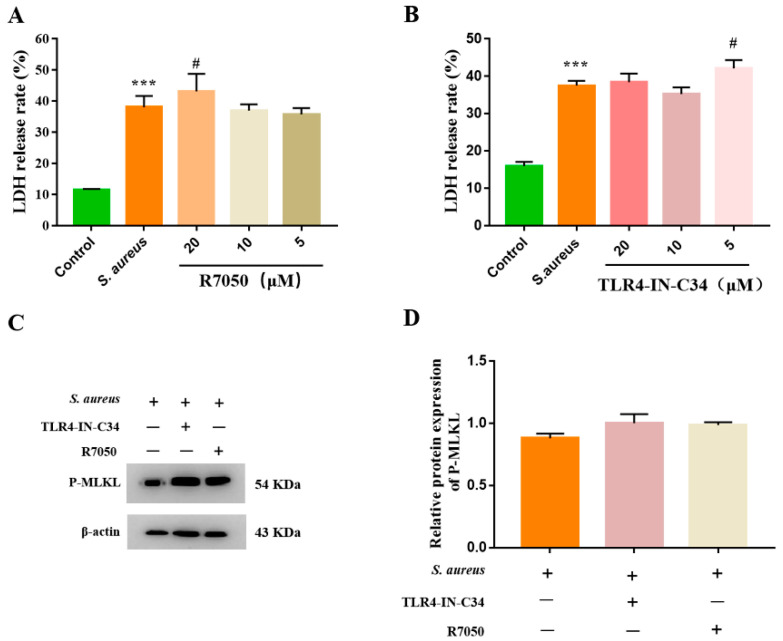
The death receptor signaling and TLR4 did not contribute to the initiation stage of gEEC necroptosis. gEECs were pretreated with the TNFR1 inhibitor (R7050) and TLR4 inhibitor (TLR4-IN-C34), respectively, for 2 h, then infected with 10 MOI *S. aureus* for 4 h. (**A**,**B**) The cytotoxicity was determined using a CytoTox96^®^ non-radioactive cytotoxicity assay kit. (**C**) Western blot analysis of P-MLKL. (**D**) Protein abundance of P-MLKL, normalized with β-actin. *** *p* < 0.001 vs. control group, # *p* < 0.05 vs. *S. aureus* group.

Several studies have demonstrated that the initiation stage of necroptosis is mediated by the signaling via death receptors, such as TNFR1 and TNFR2. In addition, TLR4 has also been shown to trigger necrosis [10]. To corroborate the dependence of *S. aureus*-induced gEEC necroptosis on the TNFR1 and TLR4, the cells were infected with *S. aureus* in the absence or presence of a TNFR1 inhibitor (R7050) and a TLR4 inhibitor (TLR4-IN-C34). As shown in Figure 3A,B, pretreatment with R7050 and TLR4-IN-C34 did not affect the LDH level in *S. aureus*-infected gEECs. Furthermore, the western blot analysis showed that pretreatment with R7050 and TLR4-IN-C34 had no apparent differences in the protein level of P-MLKL in *S. aureus*-infected gEECs (Figure 3C,D and Figure S2). Taken together, these data indicate that the gEEC necroptosis induced by *S. aureus* is not dependent on death receptor signaling and TLRs.

### 3.4. S. aureus-Induced gEEC Necroptosis Is Triggered by Ion Dysregulation and Membrane Disruption

**Figure 4:**
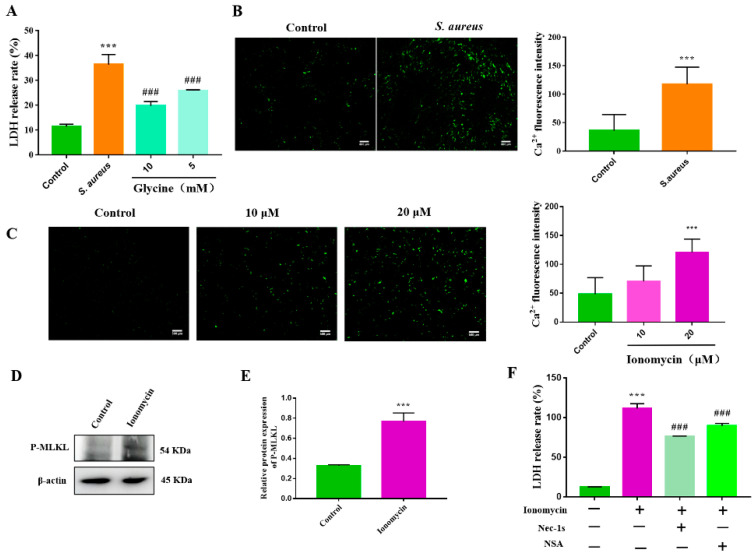
gEEC necroptosis was triggered by ion dysregulation and membranes disruption induced by *S. aureus*. (**A**) gEECs were pretreated with glycine for 2 h, then infected with 10 MOI *S. aureus* for 4 h. A CytoTox96^®^ non-radioactive cytotoxicity assay kit was used to determine the cytotoxicity. (**B**,**C**) gEECs were infected with 10 MOI *S. aureus* for 4 h or treated with ionomycin for 4 h, followed by staining with Fluo-4 AM. The image was captured using a fluorescence microscope. Scale bar: 100 μm. The Fluo-4 AM fluorescence intensity showed in right. (**D**) Western blot analysis of P-MLKL in ionomycin-treated gEECs. (**E**) Protein abundance of P-MLKL, normalized with β-actin. (**F**) gEECs were pretreated with a RIPK1 inhibitor (Nec-1s) or MLKL inhibitor (NSA) for 2 h, then treated with ionomycin for 4 h, followed by the cytotoxicity detection. *** *p* < 0.001 vs. control group, ### *p* < 0.001 vs. *S. aureus* group.

Previous studies have demonstrated that membrane disruption and ion flux dysregulation induced by the PFTs secreted by bacteria lead to the initiation of necroptosis [17,18]. *S. aureus* exerts its cytotoxic effect mainly through the secretion of toxins, such as α-toxin (Hla), a typical PFT, which disrupts the integrity of cell membranes and modulates extracellular Ca^2+^ influx [22,23]. To investigate the role of ion dysregulation in *S. aureus*-induced gEEC necroptosis, we first confirmed that *S. aureus* caused a significant increase in intracellular Ca^2+^ in gEECs, as indicated by the increase of Fluo-4 AM fluorescence intensity (Figure 4B). The fluorescence intensity was consistent with that induced by 20 μM ionomycin, which selectively induces Ca^2+^ influx (Figure 4C). Moreover, as shown in Figure 4D,E and Figure S3, P-MLKL expression was significantly increased in ionomycin-treated gEECs. Furthermore, the RIPK1 inhibitor (Nec-1s) and MLKL inhibitor (NSA) markedly reduced ionomycin-induced increase in LDH levels (Figure 4F). Importantly, glycine, which blocks cell membrane permeabilization, significantly decreased the rate of LDH release in *S. aureus*-infected gEECs (Figure 4A). These data demonstrated that membrane disruption and ion loss contribute to the initiation of *S. aureus*-induced gEEC necroptosis.

### 3.5. Necroptosis Exacerbates the S. aureus-Induced gEEC Mitochondrial Damage and ROS Generation

**Figure 5:**
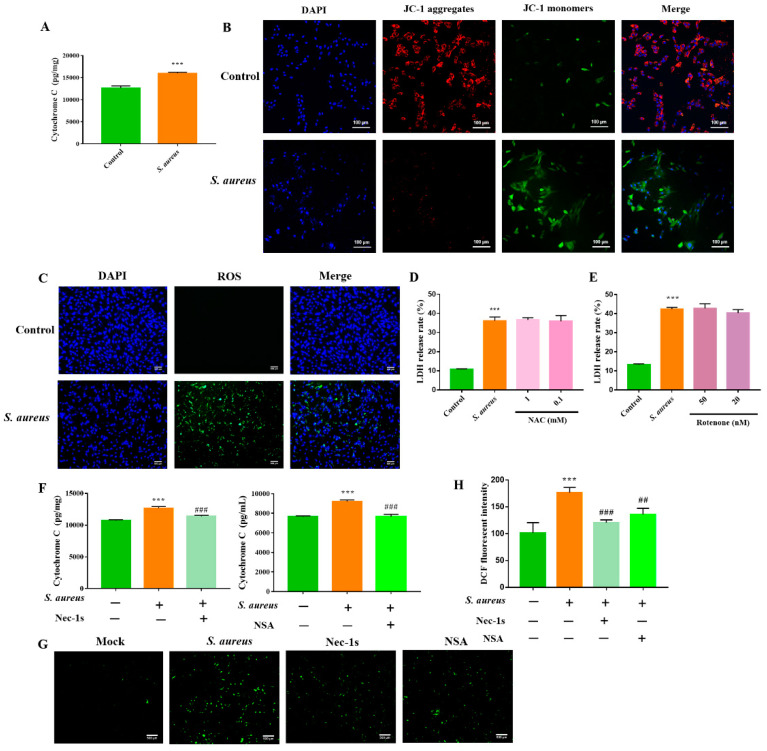
gEEC necroptosis aggravated the mitochondrial damage and ROS generation induced by *S. aureus*. (**A**) gEECs were infected with 10 MOI *S. aureus* for 4 h, then lysed cells, followed by cytochrome C levels detection using the ELISA assay. (**B**) After infection with *S. aureus* for 4 h, mitochondrial membrane potential was determined by JC-1 staining. Red represents the J-aggregates and green represents the monomer. (**C**) Fluorescence microscope analysis of ROS-DCFHDA was performed to detect the generation of ROS in *S. aureus*-infected gEECs. (**D**,**E**) gEECs were pretreated with the intracellular ROS inhibitor (NAC) and mitochondrial ROS inhibitor (rotenone), respectively, for 2 h, and then infected with 10 MOI *S. aureus* for 4 h. Then the release of LDH was detected using a CytoTox96^®^ non-radioactive cytotoxicity assay kit. (**F**,**G**) gEECs were pretreated with a RIPK1 inhibitor (Nec-1s), RIPK3 inhibitor (GSK’872), and MLKL inhibitor (NSA) for 2 h, then infected with *S. aureus* for 4 h, followed by cytochrome C and ROS detection with the ELISA assay and a ROS assay kit. (**H**) The DCF fluorescent intensity. *** *p*< 0.001 vs. control group, ## *p* < 0.01, ### *p* < 0.001 vs. *S. aureus* group.

To date, the roles of mitochondrial damage and ROS generation in host cell necroptosis induced by *S. aureus* remain unclear. To investigate the relationship between mitochondrial damage and necroptosis in gEECs, we infected cells with *S. aureus* (10 MOI) for 4 h prior to mitochondrial function analysis. As shown in Figure 5A, gEECs infected with *S. aureus* had increased levels of cytochrome C, an indicator of mitochondrial damage. Decreased mitochondrial membrane potential was also observed in *S. aureus*-infected gEECs, as indicated by increased JC-1 monomer staining (Figure 5B). In accordance with these findings, *S. aureus* promoted the ROS generation in infected gEECs, as indicated by the increased DCF fluorescence intensity (Figure 5C). These findings indicated marked mitochondrial damage and ROS generation in *S. aureus*-infected gEECs. However, *S. aureus*-induced gEEC death was not further inhibited by the blockade of the generation of mitochondrial-derived ROS with rotenone, and the presence of the ROS scavenger NAC (Figure 5D,E). 

Subsequently, to characterize the contribution of necroptosis to *S. aureus*-induced gEEC mitochondrial damage, we treated gEECs with Nec-1s and NSA before infection with *S. aureus*. As shown in Figure 5F–H, the levels of cytochrome C and ROS induced by infection with *S. aureus* were significantly decreased by this pretreatment. Thus, our collective results indicate that the necroptosis contributes to the *S. aureus*-induced gEEC mitochondrial damage and ROS generation.

## 4. Discussion

A histopathologic examination of postpartum endometritis in animals revealed necrosis and exfoliation in the endometrial epithelial layer [24,25], resulting in the impairment of innate immunity and the physical barrier functions of EECs, further increasing the risk of endometritis. Apoptosis and pyroptosis are shown to be involved in the regulation of inflammatory responses to multiple pathogenic infections [26]. It is well-known that necroptosis is a modifiable form of cell death that is involved in immune responses induced by a variety of pathogens; however, the effect of necroptosis on endometritis remains unclear. In the current study, we first confirmed that necrotic death of gEECs induced by *S. aureus* is regulated via the RIPK1/RIPK3/MLKL pathway in a process also known as necroptosis. In addition, we demonstrated that the cell death receptors did not regulate gEEC necroptosis, while Ca^2+^ influx was identified as an upstream triggering pathway. Moreover, we found that *S. aureus*-induced necroptosis exacerbated mitochondrial damage and ROS generation in gEECs. These findings advance our understanding of the molecular signaling underlying necroptosis in response to EEC injury.

Accumulating evidence indicates that a bacterial infection induces host cell necroptosis. González-Juarbe et al. demonstrated that alveolar macrophages exposed to *Serratia marcescens*, *S. aureus*, *Streptococcus pneumoniae*, or *Listeria monocytogenes* undergo necroptosis, and that inhibition of necroptosis by various means is protective against hemorrhagic pneumonia caused by *Serratia marcescens* [17]. Moreover, Kitur et al. found that Hla produced by *S. aureus* activated necroptosis mediated via the RIPK1/RIPK3/MLKL axis as a major cause of lung pathology in *S. aureus* pneumonia [15]. Despite all of these studies, the effect of necroptosis in EECs and the underlying mechanism are not well understood. In our study, the release of LDH, the PI-positive rate, and the changes in cell morphology observed by TEM provided evidence that *S. aureus* causes significant necrotic cell death in gEECs. We further showed that *S. aureus* infection upregulated the expression and phosphorylation of RIPK1 and RIPK3 proteins in gEECs, thereby promoting the formation of the necrosome, which further initiates downstream signaling, resulting in necroptosis [27]. Necrosome-mediated phosphorylation of MLKL results in MLKL oligomerization, followed by translocation to the plasma membrane and pore formation, which disrupts the integrity of the cell membrane [28]. We demonstrate that the increased expression of P-MLKL in *S. aureus*-infected gEECs was predominantly localized to the cell membrane. These provide evidence that *S. aureus* induces RIPK1/RIPK3/MLKL-dependent necroptosis in gEECs. As expected, pretreatment with the pharmacological inhibitors of RIPK1, RIPK3, and MLKL (Nec-1s, GSK’872, and NSA), respectively, partially restored the increase in the LDH release rate from gEECs induced by *S. aureus*. These data provide evidence of the involvement of necroptosis in gEEC death induced by *S. aureus* infection, although the contributions of the other major modes of cell death must be further explored.

The initial protection against pathogens in the reproductive tract endometrium relies on the innate immune system, with pattern recognition receptors (PRRs), including TLRs, NLRs, and RIG-I-like receptors (RLRs), as the first line of defense. EECs express TLRs 1–7 and 9 [29] and previous studies have shown that necroptosis is initiated by immune ligands, such as TNF, or following TLR engagement in the presence of caspase-8 inhibitors. Xia et al. showed that necroptosis could be initiated by TNFR [30]. Samson et al. also demonstrated that necroptosis is mediated by TNF-induced upregulation of RIPK1, RIPK3, and MLKL [31]. TLR and TNF-α also trigger necroptosis in pathogen-infected host cells. He et al. reported that macrophages undergo programmed necrosis via the RIPK3-dependent pathway, which is activated by TLRs [32]. Roca et al. demonstrated that *Mycobacterium tuberculosis* (MTB) infection caused the excessive release of TNF-α, resulting in the activation of the RIPK1/RIPK3/MLKL-dependent necrotic signaling pathway and producing large amounts of ROS to eliminate and control the growth and reproduction of the intracellular bacteria in macrophages [33]. Surprisingly, González-Juarbe et al. showed that lung epithelial cells undergo necroptosis following exposure to the nosocomial pathogen *Serratia marcescens* not dependent on TNFR1/2 or TLR4 signaling [18]. These studies showed that the effects of TLR or TNFR-initiated necroptosis are cell type dependent. In the current study, inhibition of TNFR1 or TLR4 did not alleviate *S. aureus*-induced gEEC necroptosis, which is consistent with the findings reported by González-Juarbe [18]. Therefore, these findings indicate that TNFR signaling and TLR4 do not participate in the gEEC necroptosis induced by *S. aureus*. These data also further defined the different mechanisms of necroptosis induced in immune cells and epithelial cells by bacterial infection and indicate that these differences may be due to the microenvironmental condition.

A bacterial infection can cause a cell membrane rupture mediated via the virulence factor PFTs, leading to an intracellular ion imbalance, which is widely reported to be a key factor in triggering necroptosis [34]. Zhu et al. showed that the increased RIPK3 level in augmented cardiomyocytes necroptosis was accompanied by an increase in intracellular Ca^2+^ levels and xanthine oxidase expression [35]. González-Juarbe et al. reported that ion dysregulation is the main trigger of necroptosis in lung epithelial cells and alveolar macrophages during bacterial pneumonia, indicating that bacterial pathogens produce the PFTs that cause membrane permeabilization and ion dysregulation, which induces activation of the necroptosis pathway in different cell types [17,18]. We also investigated the role of ion dysregulation in *S. aureus*-induced gEEC necroptosis. Pretreatment of gEECs with the selective for Ca^2+^ intake inhibitor ionomycin resulted in increased intracellular Ca^2+^ levels in a manner similar to that observed in *S. aureus*-infected gEECs. Moreover, P-MLKL expression was also increased in ionomycin-treated gEECs, and inhibition of RIPK1 and MLKL by pretreatment with Nec-1s and NSA, respectively, attenuated the gEEC death caused by ionomycin. Collectively, these results provide evidence that dysregulated Ca^2+^ influx triggers the necroptosis pathway in *S. aureus*-infected gEECs. Furthermore, the blockade of membrane permeabilization with glycine protected gEECs from *S. aureus*-induced death. Together, these results indicate a primary role for ion dysregulation during *S. aureus*-mediated activation of necroptosis in gEECs.

Numerous studies have demonstrated that *S. aureus* induces mitochondrial damage [36]. In support of these findings, our study revealed marked mitochondrial damage in *S. aureus*-infected gEECs, as evidenced by mitochondrial membrane potential depolarization as well as the increased release of cytochrome C and ROS generation. Mitochondria are the main sources of intracellular ROS, although excessive production causes mitochondrial damage and cell death. Furthermore, previous studies have shown a close relationship between mitochondrial damage, ROS overproduction, and apoptosis [37,38]. In recent studies, ROS has also been reported as an important factor that initiates or mediates necroptosis. Zhang et al. demonstrated that mitochondrial ROS promoted necroptosis by activating RIPK1 autophosphorylation of serine residue 161 (S161), and this specific phosphorylation was critical for the formation of the functional necrosome [39]. Basit et al. showed that mitochondrial mPTP opening and mitochondrial membrane potential depolarization stimulated an associated increase in ROS production, leading to necrotic cell death [40]. Huangfu et al. demonstrated that necroptosis induced by osthole was accompanied by excessive ROS production, and the ROS inhibitor NAC decreased osthole-induced necroptosis and growth inhibition [41]. It also showed that the upregulated RIPK3 initiated necroptosis via the mPTP opening induced by the ROS outburst [35]. Therefore, we explored the relationship between ROS generation and *S. aureus*-induced necroptosis in gEECs. We found that the inhibition of mitochondria-derived ROS production by rotenone treatment did not block the gEEC death. Furthermore, the effects of pretreatment of gEECs with the cellular ROS scavenger NAC to exclude the presence of ROS derived from other sources were consistent with the changes in LDH levels in rotenone-pretreated cells, indicating that mitochondrial damage and ROS do not contribute to the *S. aureus*-induced necroptosis of gEECs. However, *S. aureus*-induced ROS production and mitochondrial damage in gEECs were alleviated by necroptosis inhibitors. Overall, these findings imply that necroptosis contributes to mediating the mitochondrial damage and the generation of ROS induced in gEECs by *S. aureus* infection in some manner.

## 5. Conclusions

Our study revealed that *S. aureus* infection-induced gEEC necroptosis by upregulating the expressions of RIPK1, RIPK3, and MLKL; this process is dependent on ion dysregulation but not TLR and TNFR signaling. In addition, necroptosis exacerbated the mitochondrial damage and ROS generation in gEECs caused by *S. aureus*. Our findings may provide a theoretical basis for the understanding of the pathogenesis of endometrial necroptosis during bacterial infection, and implicate the necroptosis pathway as a target for clinical intervention during bacterial infections of the uterus postpartum.

## Data Availability

The data presented in this study are available upon request to the corresponding author.

## Supplementary Materials

The following supporting information can be downloaded at: https://www.mdpi.com/article/10.3390/ani12172218/s1, Figure S1: the original western blot figures of necroptosis-related proteins after *S. aureus* infection; Figure S2: the original western blot figures of P-MLKL in R7050 and TLR4-IN-C34 pre-treated cells; Figure S3: the original western blot figures of P-MLKL in ionomycin-treated cells.
