# Supplementary material for: Staphylococcus aureus-Induced Necroptosis Promotes Mitochondrial Damage in Goat Endometrial Epithelial Cells
Source: Animals (Basel). 2022 Aug 29;12(17):2218. doi: 10.3390/ani12172218 (PMC9454985; doi:10.3390/ani12172218)

Figure S1 Western blot analysis of RIPK3, P-RIPK3, MLKL, and P-MLKL

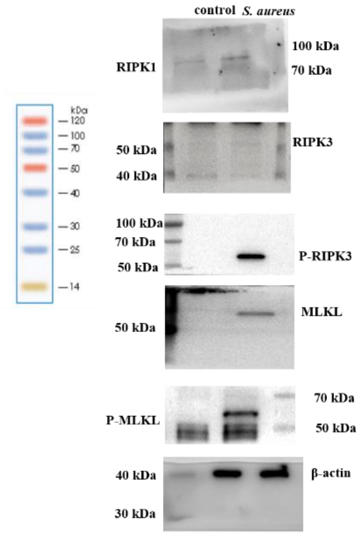

Figure S2 Western blot analysis of P-MLKL.

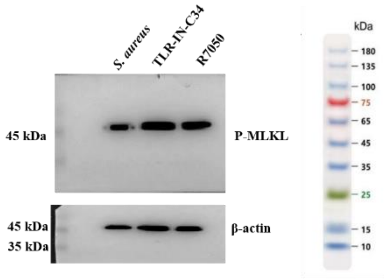

Figure S3 Western blot analysis of P-MLKL

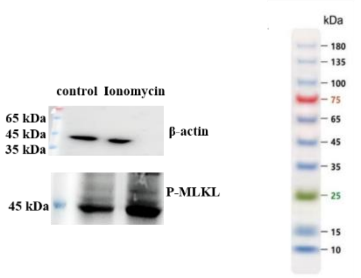

Supplement: Supplementary file 1 [file animals-12-02218-s001.zip › animals-1833978-supplementary-1.pdf]
